# Structural basis of human PRPS2 filaments

**DOI:** 10.1186/s13578-023-01037-z

**Published:** 2023-05-30

**Authors:** Guang-Ming Lu, Huan-Huan Hu, Chia-Chun Chang, Jiale Zhong, Xian Zhou, Chen-Jun Guo, Tianyi Zhang, Yi-Lan Li, Boqi Yin, Ji-Long Liu

**Affiliations:** 1 School of Life Science and Technology, ShanghaiTech University, Shanghai, 201210 China; 2 Department of Physiology, Anatomy and Genetics, University of Oxford, Oxford, OX1 3PT UK

**Keywords:** PRPS, PRPP, Cytoophidium, hPRPS2, Cryo-EM, Allosteric regulation

## Abstract

**Background:**

PRPP synthase (PRPS) transfers the pyrophosphate groups from ATP to ribose-5-phosphate to produce 5-phosphate ribose-1-pyrophosphate (PRPP), a key intermediate in the biosynthesis of several metabolites including nucleotides, dinucleotides and some amino acids. There are three PRPS isoforms encoded in human genome. While human PRPS1 (hPRPS1) and human PRPS2 (hPRPS2) are expressed in most tissues, human PRPS3 (hPRPS3) is exclusively expressed in testis. Although hPRPS1 and hPRPS2 share 95% sequence identity, hPRPS2 has been shown to be less sensitive to allosteric inhibition and specifically upregulated in certain cancers in the translational level. Recent studies demonstrate that PRPS can form a subcellular compartment termed the cytoophidium in multiple organisms across prokaryotes and eukaryotes. Forming filaments and cytoophidia is considered as a distinctive mechanism involving the polymerization of the protein. Previously we solved the filament structures of *Escherichia coli* PRPS (ecPRPS) using cryo-electron microscopy (cryo-EM) ^1^.

**Results:**

Order to investigate the function and molecular mechanism of hPRPS2 polymerization, here we solve the polymer structure of hPRPS2 at 3.08 Å resolution. hPRPS2 hexamers stack into polymers in the conditions with the allosteric/competitive inhibitor ADP. The binding modes of ADP at the canonical allosteric site and at the catalytic active site are clearly determined. A point mutation disrupting the inter-hexamer interaction prevents hPRPS2 polymerization and results in significantly reduced catalytic activity.

**Conclusion:**

Findings suggest that the regulation of hPRPS2 polymer is distinct from *ec*PRPS polymer and provide new insights to the regulation of hPRPS2 with structural basis.

**Supplementary Information:**

The online version contains supplementary material available at 10.1186/s13578-023-01037-z.

## Introduction

The pyrophosphate groups from ATP are transferred to ribose-5-phosphate (R5P) by 5-phosphate ribose-1-pyrophosphate (PRPP) synthase (PRPS) to produce PRPP [2]. PRPP is important for de novo purine and pyrimidine nucleotide metabolism and salvage pathway [3]. PRPP is also used for biosynthesis of amino acids histidine and tryptophan, NAD and NADP [4, 5]. In addition, PRPP is also used for the biosynthesis of methotrexate in archaea and pentose polyphenylphosphate in *Mycobacterium tuberculosis* [6, 7]*.* PRPS is very conservative in evolution and widely exists in all three life domains, such as bacteria, archaea and eukaryotes [8–11]. In general, an organism contains at least one gene that specifies PRPS.

There are three genes encoding PRPS in humans, namely *prps1, prps2, prps3*, which respectively encode human PRPS (hPRPS) isoenzymes 1–3 [12, 13]. hPRPS can only use ATP or dATP as pyrophosphate donors, and can be regulated by inorganic phosphate Pi and ADP allosterically [14]. Human *prps1* and *prps2* are located on the X chromosome and expressed in all tissues [12]. Human *prps3* is located on chromosome 7 and only expressed in testis [13].

There are two isoforms of hPRPS2 in vivo [15]. After the 102_nd_ amino acid residue, hPRPS2-long has three more amino acid residues than hPRPS2-short. Both hRRPS1 and hPRPS2-short have 318 amino acid residues, and the similarity is 95%. Although hRRPS1 and hPRPS2 are highly similar, they still have some different properties. For example, hPRPS2 is more sensitive to thermal inactivation, the saturated concentration of substrate is higher than hPRPS1, and it is unlikely to be inhibited by nucleoside diphosphate [16].

As a rate limiting enzyme, the regulation of PRPS is very complex. hPRPS uses ATP or dATP as pyrophosphate donor and can be allosterically regulated by inorganic phosphate Pi and ADP. The activity of hPRPS depends on Pi, and inhibited by ADP [16, 17].

In the human body, hPRPS1 and hPRPS2 have different functions. When the activity of hPRPS1 mutants increases, it will cause hyperuricemia, myasthenia, gouty arthritis or neurosensory defects [18]. When the activity of hPRPS1 mutants is reduced, it will lead to neuropathy, deafness or intellectual disability [19–21]. Compared with hPRPS1, hPRPS2 plays a more important role in the occurrence and maintenance of cancer caused by the transcription factor Myc [22].

The increase of nucleotide synthesis will inhibit hPRPS1, while the inhibition of nucleotide on hPRPS2 is less obvious. Moreover, *prps2* gene has one more pyrimidine rich translation element (PRTE) in the 5 '- Un-translated region than *prps1* gene, which is controlled by the oncogene and translation initiation factor eIF4E downstream *Myc* activation. [22]. The eukaryotic initiation factor eIF4E and other factors interact with PRTE to increase the transcription of *prps2* mRNA, thereby increasing the concentration of hPRPS2 to promote the synthesis of nucleotide. hPRPS2 has a synthetic lethal effect in cells with high *My*c expression. These evidences indicate that hPRPS2 plays an important role in the metabolism of cancer cells with high expression of *myc* [22].

The crystal structure of PRPS shows that PRPS is assembled into hexamers [23, 24]. PRPS formed filamentous structures in a variety of eukaryotic cells such as yeast, *Drosophila* oocytes, rat neurons, human fibroblasts [25] and zebrafish retinal epithelial cells [26]. Recently, we also found that PRPS can form filamentous structures in prokaryotes such as *Escherichia coli* in vitro and in vivo [1]. We further understood the filament structure *E. coli* PRPS (ecPRPS) at near atomic resolution using cryo-electron microscopy (cryo-EM).

Here, we find that hPRPS2 can form filaments in vitro, and the structure of hPRPS2 is obtained by cryo-EM with 3.08A resolution. In this structure, we can see a clear hexamer, and we also find the key amino acid residues at the hexamer interface. A point mutation on the interface can destroy filament assembly, resulting in reduced enzyme activity. We also obtain clear ligand information of allosteric sites and active sites.

## Results

### hPRPS2 assembly into filaments

Recently, we have solved the filament structures of *E. coli* PRPS [1]. However, the structure of hPRPS2 and whether it can form filament structure are still unclear. We purified the short isoform of hPRPS2 and tried to induce polymerization in vitro under different conditions. Unlike ecPRPS, there are fewer conditions for hPRPS2 to form a filamentary structure. hPRPS2 canform filaments when incubated with ADP and Mg^2+^.(Additional file 1: Figure S1).

**Fig. 1:**
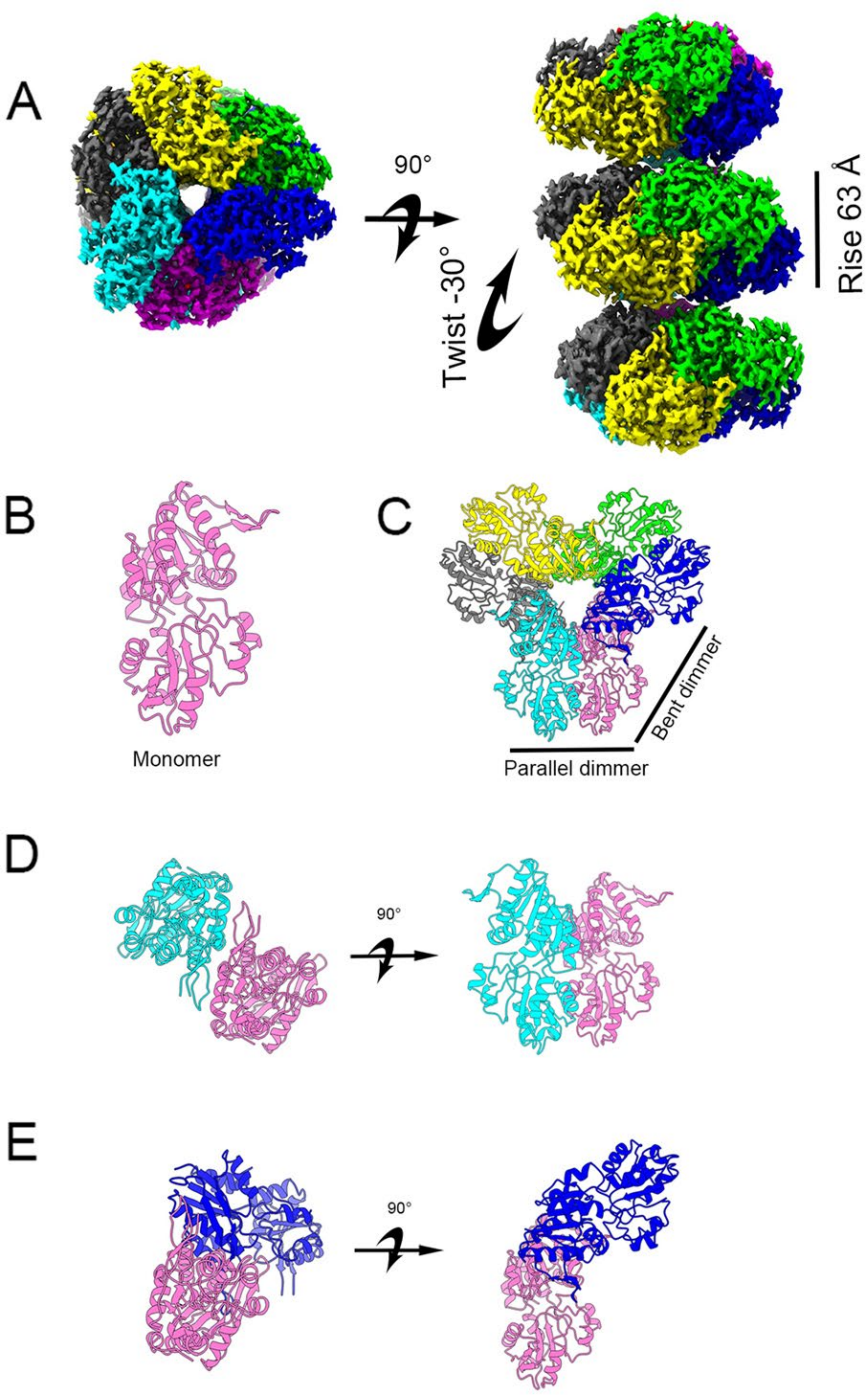
Overall structure of human PRPS2 filament. **A** The electron density map of type A filament (3.08 Å resolution) shows that the rise of human PRPS2 filament is 63 Å. When hexamers are aggregated into the filament, the adjacent hexamer twists by 30°. **B** Monomer of human PRPS2. **C** Hexamer of human PRPS2. **D** Parallel dimer of human PRPS2. **E** Bent dimer of human PRPS2. Each chain has a different color in **C**–**E**

Using cryo-EM and single particle analysis, we solved the filament structure of hPRPS2, with the resolution of central layer map was estimated to be 3.08 Å (Fig. 1A; Additional file 2: Figure S2). In the reconstructed model, hPRPS2 also forms a hexamer structure, similar to hPRPS1 or other PRPS (Fig. 1B–D, Additional file 3: Figure S3). Hexamers are the basic unit of filament polymerization (Additional file 3: Figure S3). The twist and rise of hPRPS2 filament are 30° (left-handed twist) and 63 Å, respectively (Fig. 1A). The cryo-EM data and model refinement statistics are shown in Table 1.

**Table 1:** Cryo-EM data statistics

	Human PRPS2-short filament
EM equipment	Titan Krios
Detector	K3 camera
Magnification	22,500x
Voltage (kV)	300
Electron exposure ((e–/Å^2^))	60
Defocus range(μm)	− 1.0 to − 2.5
Pixel size(Å)	1.06
Symmetry imposed	D3
Number of collected movies	2403
Initial particle images (no.)	681,672
Final particle images (no.)	140,303
**Refinement**	
EMDB ID	EMD-33883
PDB code	7YK1
Initial model used (PDB code)	-
Map resolution (Å)	3.1
FSC threshold	0.143
Map resolution range (Å)	2.9–3.9
Map sharpening B-factor(Å^2^)	-74
Model composition	
Non-hydrogen atoms	14,364
Protein residues	1836
Ligands	ADP,Pi,Mg
Ions	12
B factors(Å^2^)	
Protein	58
Ligand	67
R.m.s. deviations	
Bond lengths (Å)	0.005
Bond angles (°)	0.690
Validation	
MolProbity score	1.70
Clashscore	5.53
Poor rotamers (%)	0.13
Ramachandran plot	
Favored (%)	93.98
Allowed (%)	5.24
Disallowed (%)	0.77

### Ligand binding modes in hPRPS2 filaments

**Fig. 2:**
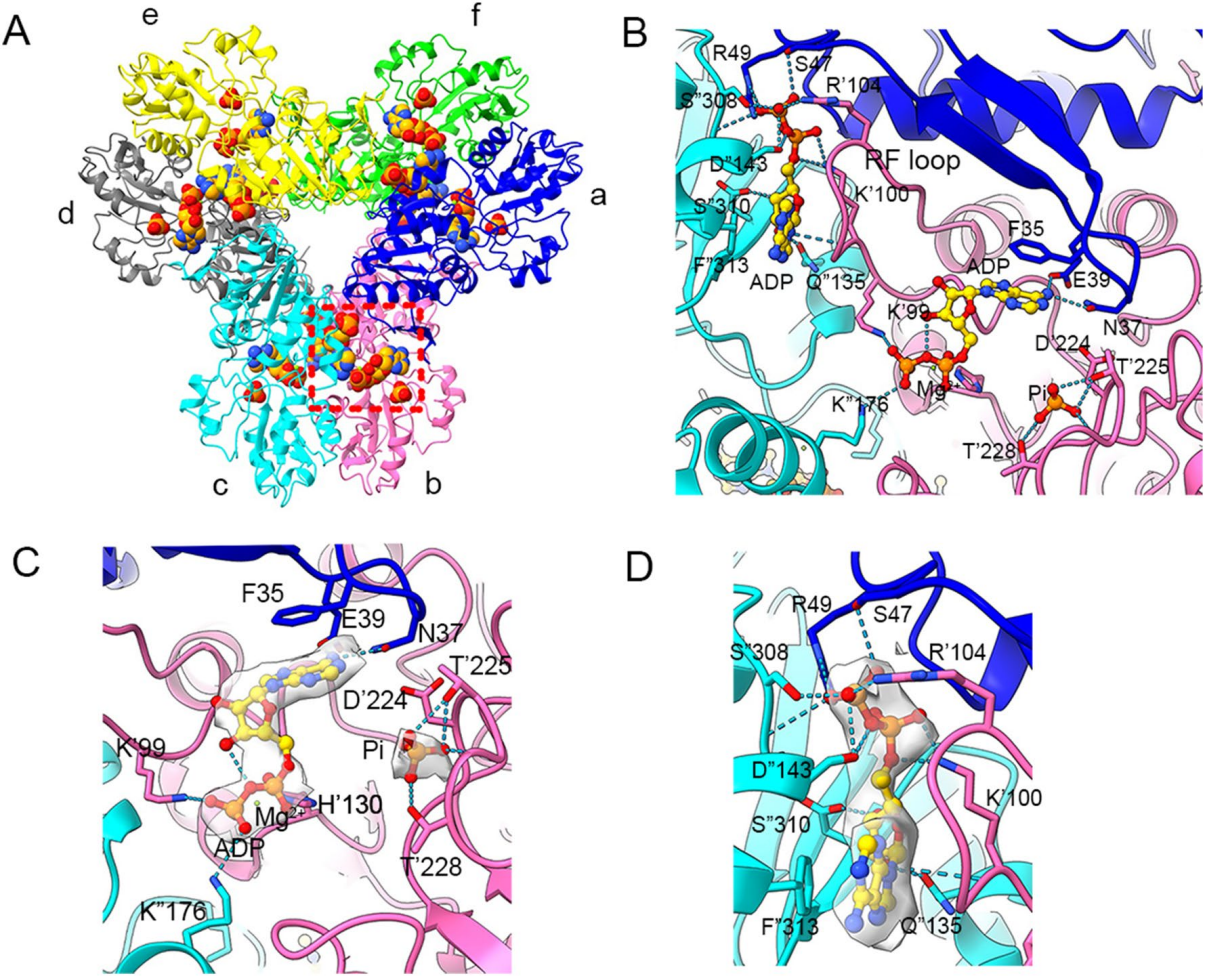
Ligand binding modes in human PRPS2 filament. **A** Hexamer of human PRPS2 filament. Each chain is marked with a different color. The areas marked in red are those shown in Figures B, C and D. **B** ADP is recognized on the allosteric site and active site of human PRPS2 filament, while R5P binding site is bound by Pi. The residues interacting with ligands are indicated. Residues in chain b are numbered with the ‘ symbol, and the residues in chain c are numbered with the “ symbol. **C** Ligands of the active site of human PRPS2 filament. ADP and Mg^2+^ occupy ATP binding sites at active sites. Pi can also be seen in the active site. Each chain is marked different colors. **D** ADP in the allosteric site of human PRPS2 filament. Each chain is marked with different colors

The hexamer of hPRPS2 had D3 symmetry, there were 6 active sits (ATP and R5P binding site)and 6 allosteric sites in a hPRPS2 hexamer. ADP was found at ATP binding site and allosteric site in hPRPS2 filament. There is a Pi in the phosphate binding region of R5P, which may come from the hydrolysis of ADP or be preserved during protein purification (Fig. 2A and B). Pi at the R5P active site forms hydrogen bonds with T225, T228 and the backbone of G227.

ADP at the ATP active site binds to Mg^2+^, similar to the *E.coli* PRPS type A filament structure (PDB:7XMU and 7XMV)^1^. ADP in chain b forms hydrogen bonds with N37 and E39 in chain a. There is a π-π interaction between F35 in chain a and adenine base. K99 and H130 form salt bridges with the β-phosphate and α-phosphate separately, and Mg^2+^ coupling the α- and β-phosphate of ADP with H130. K176 in chain c also forms salt bridge with β-phosphate, which is different from *E.coli* PRPS (Fig. 2C). The ADP in the active site can interact with three monomers, which means hPRPS2 needs to be assembled properly to function. ADP and Pi can also compete with substrates (ATP and R5P) to bind at the active site. The allosteric regulators ADP and Pi can also act as competitive factors to regulate enzyme activity.

ADP at allosteric site of chain cforms hydrogen bonds with S47 in chain a, and S308 and S310 in chain c by the β-phosphate. The backbone of K100 and D143, and side chain of Q135 in chain c also form hydrogen bonds with the C-1, C-2 and C-2 hydroxyl groups, respectively. In addition, there is a π-π interaction between F313 of chain c and adenine base. Salt bridges are formed between R49 in chain c, R104 in chain a with β-phosphate, and K100 in chain b with α-phosphate (Fig. 2D). ADP in allosteric site also interacts with 3 monomers, and it formed hydrogen bond with regulatory flexible loop (RF loop Y94-S108). We speculated that ADP can regulate enzyme activity through competitive inhibition and allosteric inhibition by affecting the conformation of RF loop.

### Contacts of hexamers in hPRPS2 filaments

**Fig. 3:**
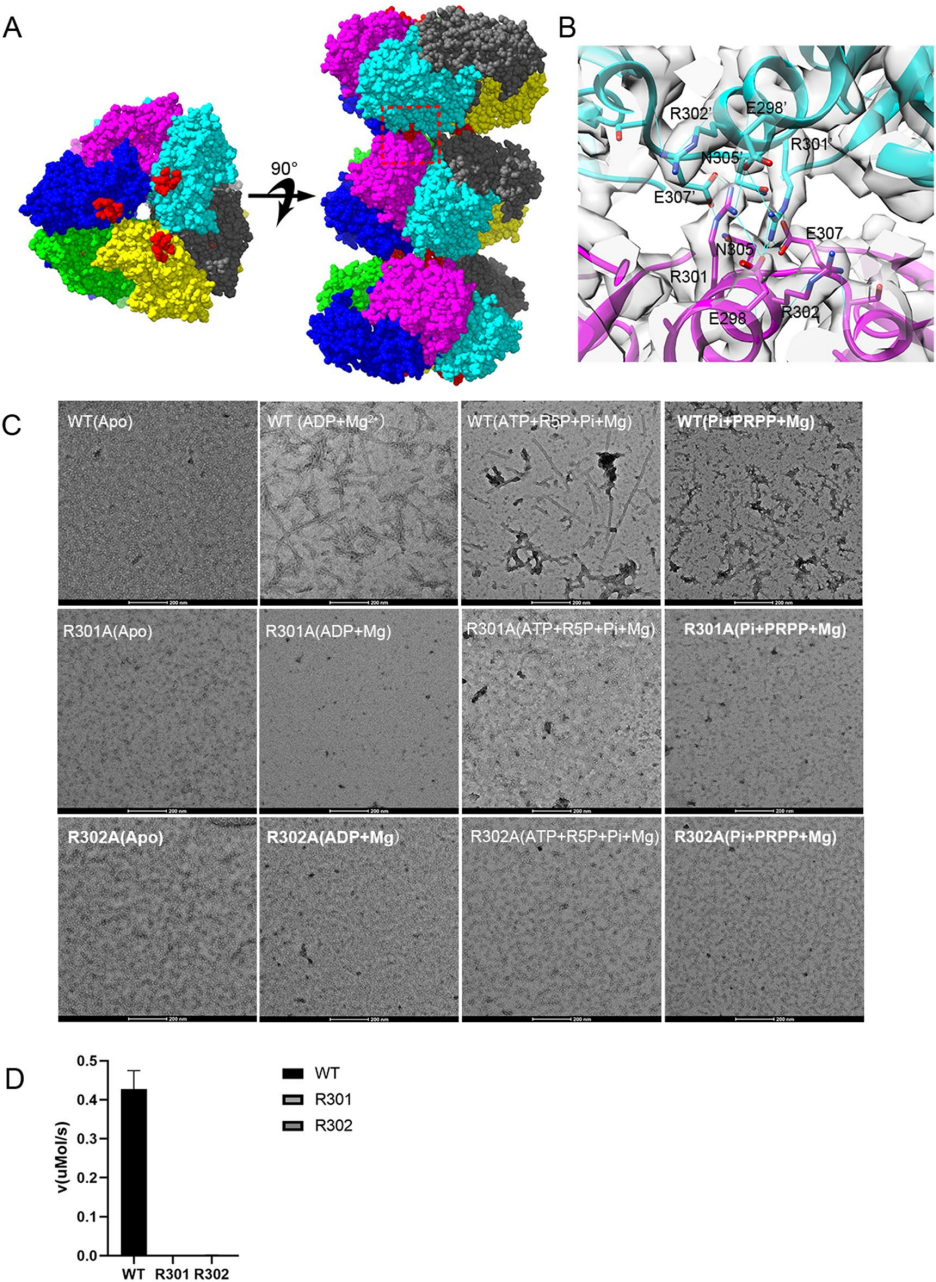
Contact of hexamer in human PRPS2 filament. **A** and **B** Maps and models of human PRPS2 filament between adjacent hexamers. The residues responsible for the interactions are indicated. The area marked in red is that shown in Figure **B**. **C** Negative staining of human PRPS filament. The wild-type human PRPS2 can form filament under ADP + Mg^2+^, ATP + R5P + Pi + Mg^2+^,or Pi + PRPP + Mg^2+^ condition. Mutant R301A and R302A can disrupt filament formation. The scale bar is 200 nm. **D** Enzyme activity assay of wild-type human PRPS2 and its mutant. Mutant R301A and R302A almost lost its activity

The filament of hPRPS2 is stacked by hexamers with D3 symmetry. There are three interaction sites between two adjacent hexamers. Each interaction site contains some identical amino acids (Fig. 3A). hRPPS2 hexamers are connected by salt bridges formed between R301 and E298 pairs, the hydrogen bonds between R301, N305, and E307, and also the van der Waals’ Forces between R302 and R301 (Fig. 3B). These 5 residues forms a complex network of interactions include π-π interactions between arginines with the same residues in the neighboring hexamer.

From our previous study on *E. coli* PRPS, we found that the residue R302 is the key amino acid for PRPS filamentation. Therefore, we generated a mutant R301A and R302A of hPRPS2. The filament forming ability was evaluated by negative staining electron microscopy. When incubated with allosteric regulator ADP and Mg^2+^, or ATP, R5P, phosphate and Mg^2+^, or PRPP, phosphate and Mg^2+^, hPRPS2 could form long filaments, and the mutant hPRPS2^R301A^ and hPRPS2^R302A^ lost its filament-forming ability (Fig. 3C).

To investigate the function of hPRPS2 filament, we used the coupling reaction method to test enzyme activity in vitro. Phosphoribosyltransferase (OPRT) can consume PRPP in the reaction orotate (OA) + PRPP → orotidine 5'-monophosphate (OMP) + PPi. OA has absorption at 295 nm, and the production of PRPP can be measured by the consumption of OA. The enzyme activity of hPRPS2 depends on the activator Pi. When the concentration of Pi was less than 10 mM, hPRPS2 did not show any activity. When Pi concentration was higher than 30 mM, hPRPS2 catalyzed the reaction at maximum velocity (Additional file 4: Figure S4). Therefore, we added 30 mM Pi reaction mixture to find the suitable substrate concentration. And we found 30 mM Pi, 1 mM ATP and 1 mM R5P were suitable concentration for enzyme activity assay.

Finally, we tested the enzyme activity using 1 mM ATP, 1 mM R5P and 30 mM Pi. The enzyme activity results showed that the mutant hPRPS2^R301A^ and hPRPS2^R302A^ almost lost its activity (Fig. 3D). These data indicate that filamentation of hPRPS2 is very important for its activity.

### Comparison of hPRPS1 and hPRPS2 hexamers

**Fig. 4:**
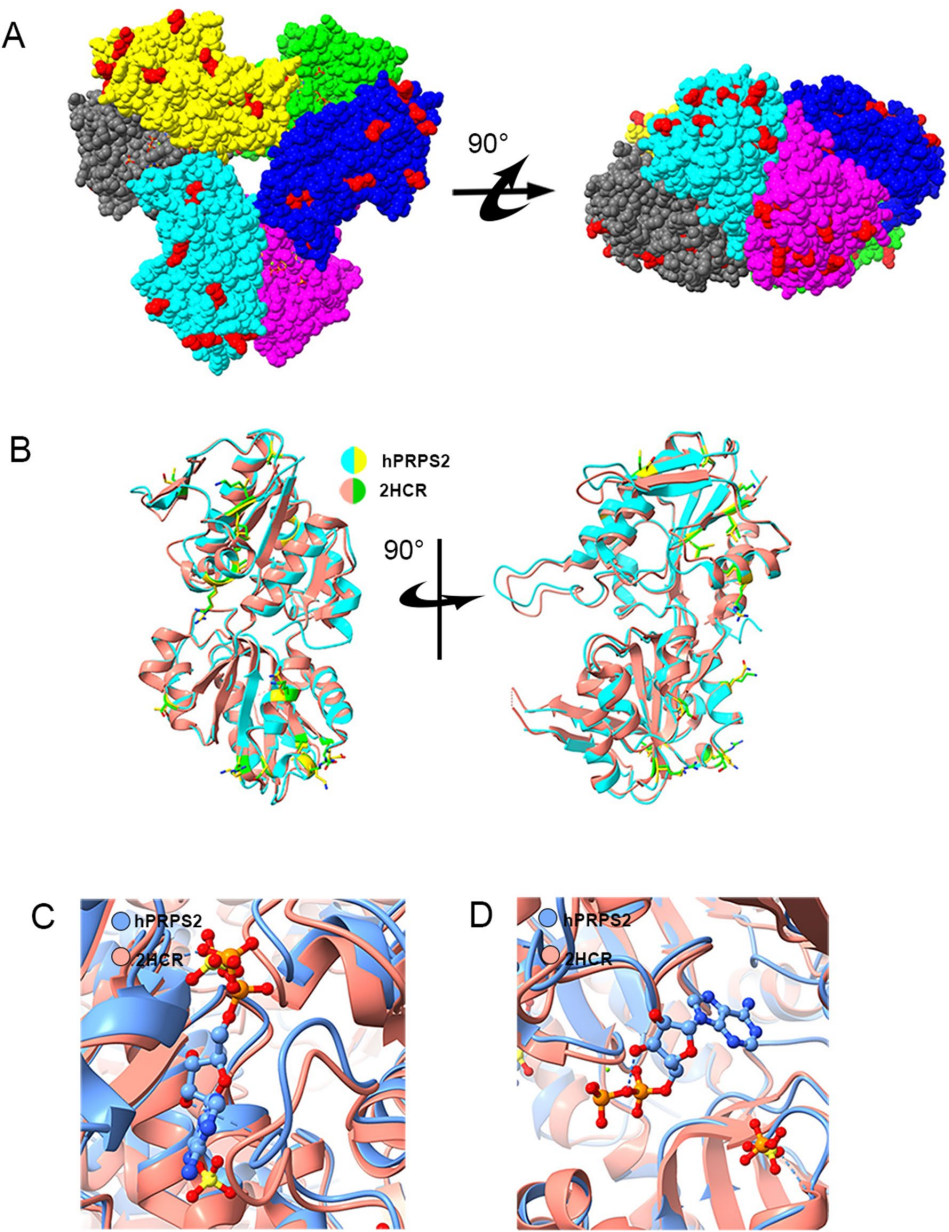
Structural comparison of hPRPS2 and hPRPS1 (2HCR). **A** The difference of amino acids between hPRPS1 (2HCR) and hPRPS2. There are 15 amino acid residues differences between human PRPS1 monomer and human PRPS2 monomer. Most of the different amino acids are located on the surface of the hexamer. The amino acid residues with differences are marked in red. **B** Comparison of hPRPS1 and hPRPS2 monomers. The monomer of human PRPS1 is in red and human PRPS2 is in cyan. The amino acid residues with difference in human PRPS1 and human PRPS2 are green and yellow, respectively. **C** Comparison of allosteric site and RF loop. There is an ADP at the allosteric site of human PRPS2, while SO_4_^2−^ in human PRPS1 can bind to allosteric site and another site. The chain of human PRPS1 (2HCR) is in red and of human PRPS2 is in blue. **D** Comparison of human PRPS1 (2HCR) and human PRPS2 active sites. In human PRPS2, ADP and magnesium occupy the ATP binding site in the active site, which is empty in the ATP binding site of human PRPS1 (2HCR). SO_4_^2−^ and PO_4_^3−^ are found in the R5P binding sites of human PRPS1 (2HCR) and human PRPS2, respectively

We compared the sequences of hPRPS1 and hPRPS2, and found that they had only 15 amino acid residues differences. We labeled these different amino acid residues in the structure of hPRPS2 hexamer (Fig. 4A). Almost all the different amino acid residues are located on the surface of the hexamer. They are far away from active sites, allosteric sites and hexamer-hexamer interaction sites.

Some crystal structures of hRPPS1 and its mutants have been solved. We compared the crystal structures of wild-type hPRPS1 (2HCR) and our cryo-EM structure of hPRPS2. We labeled the amino acids with differences in the matched monomer of hPRPS2 and hPRPS1 (2HCR). All these amino acids are located at the same position (Fig. 4B). The monomer of hPRPS1 and hPRPS2 are highly similar, which may indicate that hPRPS1 and hPRPS2 can form heterohexamers.

The structure of hPRPS2 is very similar to that of hPRPS1 (2HCR). In the structure of hPRPS1 (2HCR), SO_4_^2−^ occupies the β-phosphate of ADP, and another SO_4_^2−^ binds to the second regulator site (Fig. 4C). From this structure, ADP can also bind to the allosteric site, and the binding of ADP will block the phosphate binding of allosteric sites. The active sites of hPRPS2 and hPRPS1 have little change. Both phosphate and SO_4_^2−^ can bind to the R5P binding site of the active site (Fig. 4D). The allosteric site and active site of hPRPS1 and hPRPS2 short isoform are highly conserved and similar, which may indicate that they have the same regulation mode.

### Comparison of hexamer interfaces in human and *E. coli* PRPS

**Fig. 5:**
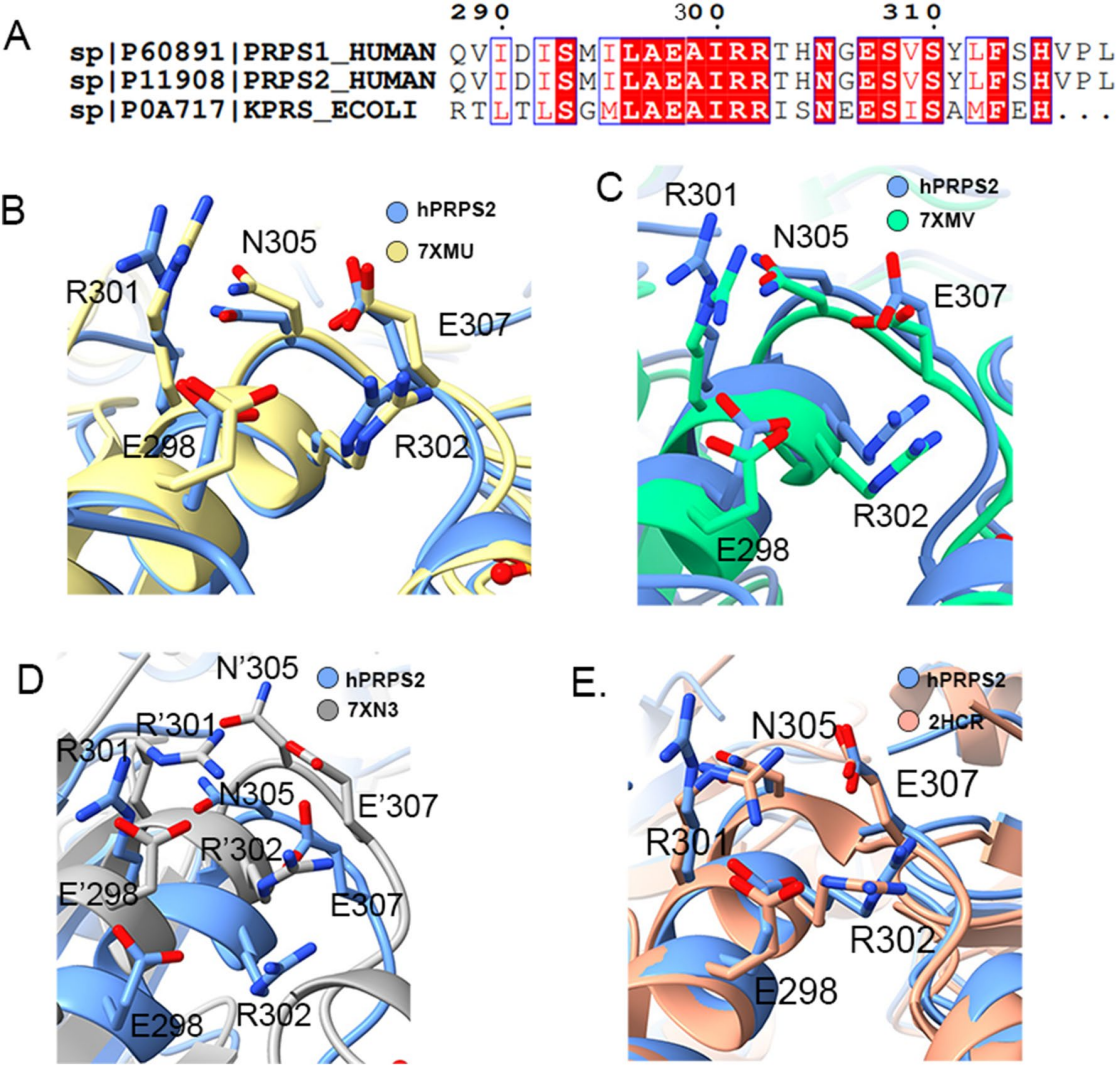
Structural comparison of interfacial amino acid residues in different organisms. **A** Sequence alignment of interfacial amino acid residues in different organisms. Some amino acid residues at the interface of two adjacent hexamers in human PRPS2 filament are conserved. The conserved amino acid residues are shown in red. **B** Structural comparison of interfacial amino acid residues between hPRPS2 (blue) and *E. coli* type A filament PRPS (7XMU) (yellow). The human PRPS2 filament is the same as *E. coli* PRPS type A filament, and the amino acid residues involved in the hexamer interconnection are conserved. **C** Structural comparison of interface amino acid residues between human PRPS2 (blue) and *E. coli* type A^ADP+AMP^ filament PRPS (7XMV) (green). The position of the amino acids involved in the interconnection of hexamers are highly similar. **D** Structural comparison of interface amino acid residues between human PRPS2 (blue) and *E. coli* type B filament PRPS (7XN3) (gray). Compared with *E. coli* type B filament PRPS (7XN3), the position of amino acid residues involved in the interconnection of hexamers has shifted. The amino acid residues in *E. coli* type B filament PRPS (7XN3) are labeled with ‘. (**E**) Structural comparison of interface amino acid residues between hPRPS2 and hPRPS (2HCR). The amino acid residues in hPRPS (2HCR) are labeled with ‘

According to sequence alignment, we found that the amino acid residues connecting two adjacent hexamers were highly conserved in hPRPS and *E. coli* PRPS (Fig. 5A). The hPRPS2 filament is similar to *E. coli* PRPS type A (7XMU) and type A^ADP+AMP^ (7XMV) filaments (Additional file 5: Figure S5). They have the same amino acid residues connecting adjacent hexamers.

The positions of amino acid residues E298, R301, R302, N305 and E307 in the structures of hPRPS2 and *E. coli* PRPS type A filament (7XMU) and type A^ADP+AMP^ (7XMV) filament did not changed significantly (Fig. 5B and C). hPRPS2 and *E.coli* PRPS can form filament, and their key amino acid residues for filamentation were highly conserved. This shows that the formation of filament structures is very important and conserved for the function of prokaryotes and eukaryotes.

*E.coli* PRPS type B filament has another interface between adjacent hexamers. Y24 is the key amino acid residue of *E.coli* PRPS type B filament. The residue Y24 is not conserved in hPRPS1 and hPRPS2. Our study did not find hPRPS2 can form another type of filament. There is a greater difference between hPRPS2 and *E. coli* type B filament (7XN3), the amino acid residues related to the formation of hPRPS2 filament have a great displacement relative to *E. coli* type B filament (Fig. 5D).

We also compared the crystal structures of hRPPS1 (2HCR) and our hRPPS2 cryo-EM structure, which have very similar structures at the interface between two adjacent hexamers of the filament (Fig. 5E). hPRPS1 and hPRPS2 have the same amino acid residues critical for filamentation. We speculate that hPRPS1 and hPRPS2 can be assembled into a mixed filament.

### The RF loop of hPRPS2

**Fig. 6:**
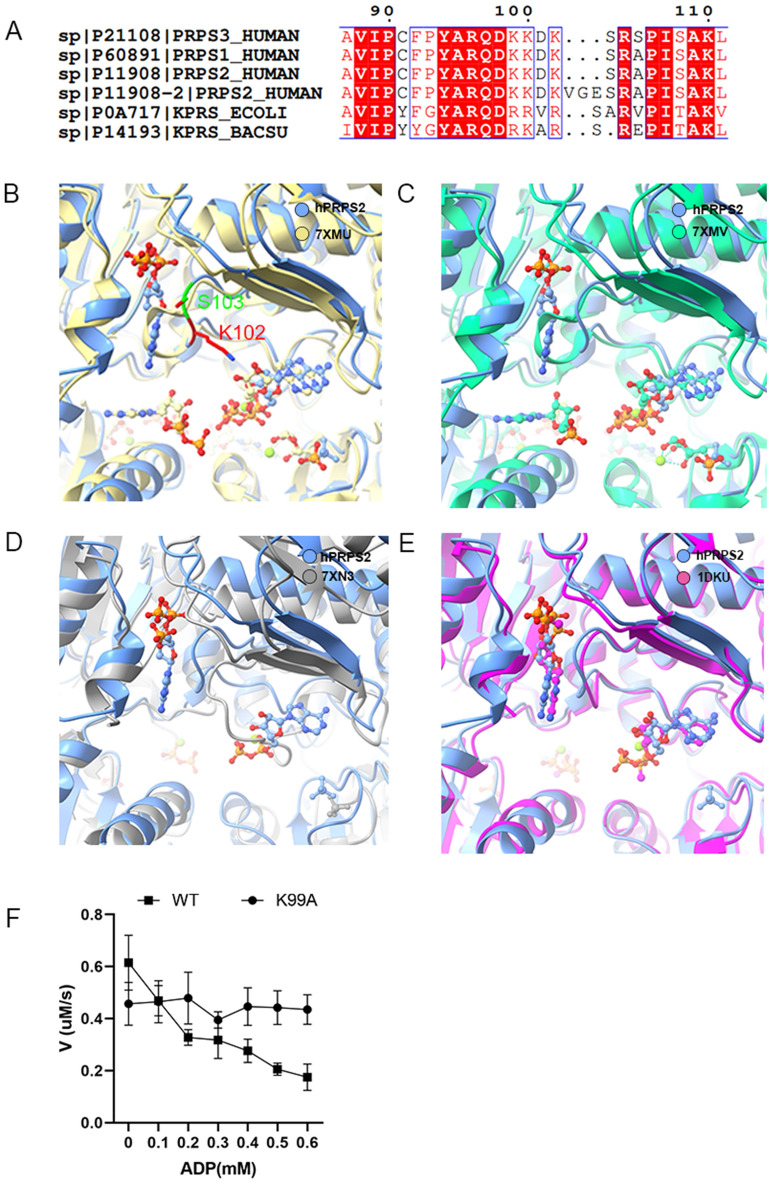
Comparison of PRPS ligands in different organisms. **A** The sequence alignment of RF loop of hPRPS1-3, *E. coli* PRPS and *Bacillus subtilis* PRPS. Structural comparison between human PRPS2 with *E. coli* PRPS in type A filament (B: 7XMU), *E. coli* PRPS in type A^ADP+AMP^ filament (C: 7XMV), *E. coli* PRPS in type B filament (D: 7XN3), and *Bacillus subtilis* PRPS (E: 1DKU). In (**B**) and (**C**), ADP in allosteric site 1 clashes with the RF loop in *E. coli* PRPS. The difference of the RF loop in hPRPS2 short isoform is indicated (K102 and S103) in (**B**). In (**D**), ADP binds to the ATP active site of hPRPS2. Unlike *E. coli* type B filament, RF loop of hPRPS2 does not occupy the ATP site. In (**E**), the RF loop of human PRPS2 is slightly different from that of *Bacillus subtilis* PRPS. (**F**) The results of ADP inhibition on hPRPS2-short^wt^ and hPRPS2-short^k99A^. The activity of hPRPS2-short^k99A^ is low, but not inhibited by ADP

The RF loop of hPRPS2 is located between the active site and the allosteric site. There are two isoforms of hPRPS2: hPRPS2-long and hPRPS2-short. hPRPS2-long has three residues insertion after residue K102. Sequence alignment showed that the RF loop was very conserved in bacteria and human. In the compared organisms, only *E. coli* PRPS has an Alanine (A) after S103 in the RF loop (Fig. 6A). This study solved the short isoform PRPS2 structure.

When comparing the structure of hPRPS2 with that of *E. coli* type A (7XMU) and type A^ADP+AMP^ (7XMV) filaments, the regulatory flexible loop (RF loop Y94-T109) of *E. coli* clashes with ADP in human PRPS2 allosteric site 1. The RF loop conformation of hPRPS2 (Y94-S108) is very similar to that of *E. coli* PRPS including loops Y94-D101 and R104-S108 (Y94-V101 and R105-T109 in *E. coli*).

The loop K102-S103 (R102-S103-A104 in *E. coli*) of hPRPS2 is shorter than that in *E. coli* and deviates from the *E. coli* loop by about 4.7 Å. Structure comparision between hPRPS2 and *E.coli* PRPS type A filaments (7XMU, 7XMV) showed that the RF loop of *E.coli* PRPS can hinder ADP bind to allosteric site(Fig. 6B and C). When comparing the structure of hPRPS2 with that of *E. coli* type B filament (7XN3), the RF loop of *E.coli* PRPS can occupy ATP sites to prevent ATP bind to the active site (Fig. 6D). This indicates that ADP binding at allosteric site 1 does not prevent ATP from entering its active site. While comparing the structure of hPRPS2 with that of *Bacillus subtilis* PRPS (1DKU), the RF loops between hPRPS2 and *Bacillus subtilis* PRPS are slightly different. When binding the same ligand, *Bacillus subtilis* PRPS may not form filament like hPRPS2 (Fig. 6E).

**Fig. 7:**
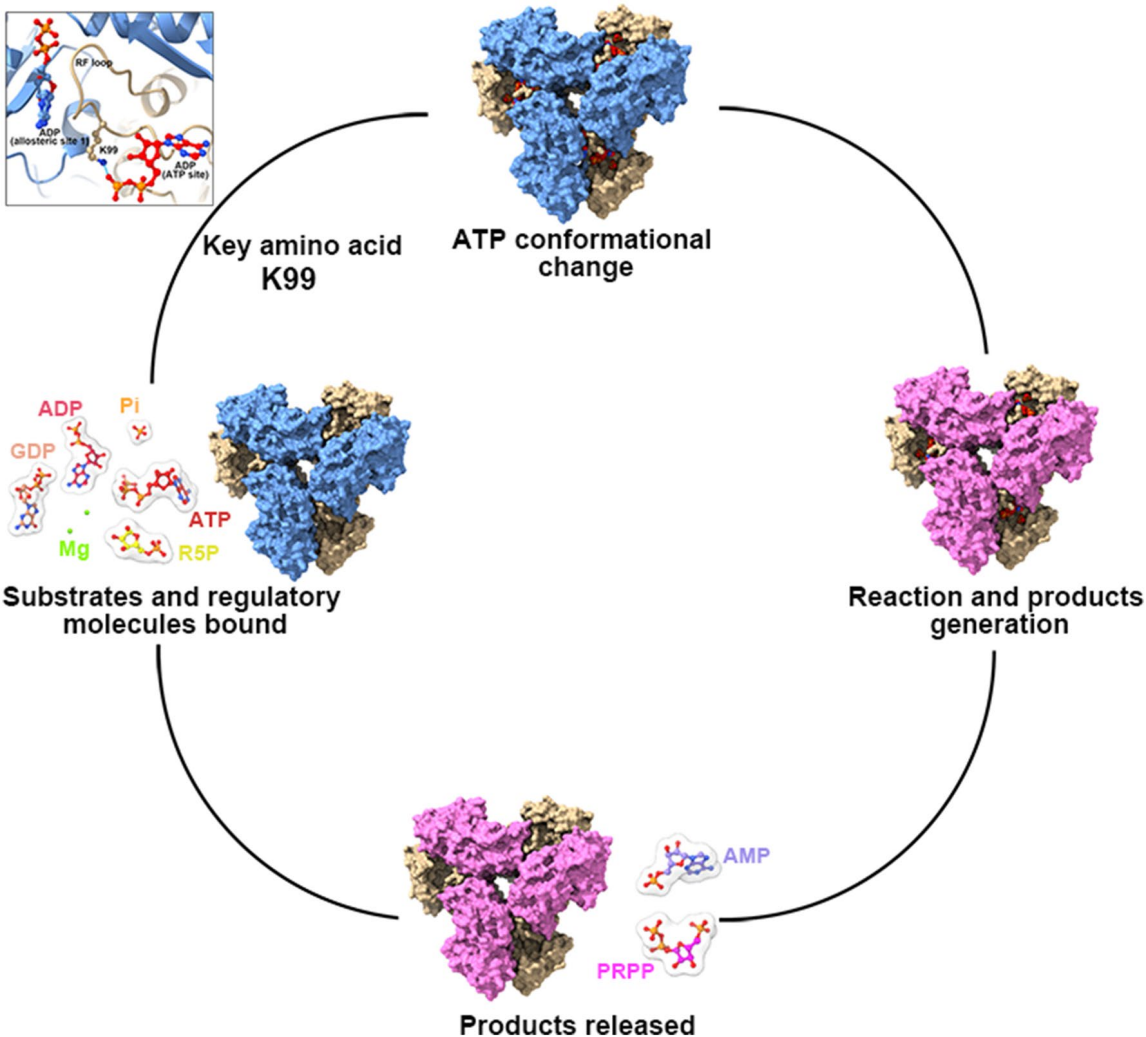
A working model of hPRPS2 regulation. The ADP of allosteric site can interact with RF loop. Residue K99 of RF loop can participate in the regulation of ATP conformation in active site. RF loop regulates the enzyme activity by directing the conformational changes of ATP

RF loop can swing back and forth between active and allosteric sites to control ligands binding. In order to study the function of RF loop, we generated a mutant K99A and tested its changes in enzyme activity characteristics. The most suitable concentration of substrates (30 mM Pi, 1 mM ATP and 1 mM R5P) were used for ADP titration. According to our model, the residue K99 located in the RF loop can interact with β-phosphate of ATP at the active site. K99 is the key amino acid for RF loop to change ATP conformation. When K99A mutation occurred, we found that the inhibition of ADP was eliminated. When there was no ADP in solution, the activity of mutant K99A was lower than that of wild-type (Fig. 6F). Therefore, we speculate that the RF loop can help ATP enter the active site and participate in the allosteric regulation of ADP (Fig. 7).

## Discussion

Previous studies of the regulation mechanisms of human PRPS mainly focused on PRPS1. Here, we solve the human PRPS2 filament structure with ADP binding in ATP site and allosteric site 1 and Pi binding in the R5P site.

### hPRPS2 and disease

PRPS2 is associated with Myc driven cancers, such as prostate cancer, neuroblastoma, osteosarcoma [27–29]. PRPS2 may be a promising diagnosis and therapy target for these cancers. In addition, PRPS2 is associated with low spermatogenesis and Sertoli cell-only syndrome (SCOS), which may be a potential biomarker and therapy target of male infertility [30, 31]. The precise regulation of PRPS is crucial to the physiological function of organisms, because both gain-of-function and loss-of-function mutations of PRPS are related to severe human disorders [19, 21, 32].

### PRPS in prokaryotes and eukaryotes

Our recent study showed that two types of ecPRPS filaments play important roles in enzyme activity regulation [1]. The ecPRPS type A filament attenuates the allosteric inhibition by ADP, while the ecPRPS type B filament may impede the binding of ATP.

Now we find hPRPS2 can also form filament in vitro, which is similar to *E. coli* PRPS type A filament. They have the same key interface amino acid residues for filament formation, which is conserved in many organisms. Although the mutants R301A and R302A in hPRPS2 almost lost activity, the mutants R302A in *E. coli* can function normally in the absence of allosteric regulator ADP. The amino acid for *E.coli* PRPS type B filament formation (Y24) was not conserved in human PRPS, so we only found hPRPS2 form filament like *E.coli* PRPS type A filament.

Previous studies also showed that PRPS could form heterogenous protein complex with a molecular weight greater than 1000 kDa from rat liver and human tissues [33]. Recent studies reported that PRPS form cytoophidia in a variety of eukaryotes including yeast, zebrafish and human [25, 26, 34].

### ADP regulation of PRPS

ADP/GDP allosteric inhibition of PRPS has become a consensus, but how ADP/GDP inhibits this enzyme is still unclear. From our model, we obtain the information of ADP binding to the allosteric site and active site. This indicates that ADP has both allosteric regulation and competitive inhibition. The RF loop (Y94-T109) is located between the active site and the allosteric site, and it can interact with ADP in the active site and the allosteric site. Structure comparison shows that the RF loop may block the binding of ADP to the allosteric site.

According to our model, although ADP binds to the allosteric site, the RF loop does not occupy the ATP binding site of the active site. On the contrary, ADP bound to the allosteric site interacts with the RF loop to keep the loop in an open conformation state, which helps ATP enter. In addition, when ADP binds to allosteric site, the residue K99 (in the RF loop) can interact with the β phosphate of the ATP (ADP in our model) at the active site. Therefore, we speculate that ADP allostricly inhibit enzyme activity is not by preventing ATP from entering the active site, but rather by interacting with RF loop to prevent ATP conformation from changing. The K99 in the RF loop played an important role in changes of ATP conformation.

Studies have shown that ATP must undergo conformational change before catalysis (Fig. 7) [35, 36]. When ADP in the allosteric site interacts with the RF loop and affects the conformation of the RF loop, the interaction between K99 and the β phosphate of ATP may prevent the conformational change of ATP and disrupt the reaction cycle. To test this idea, residue K99 is mutated. Mutant K99A is not inhibited by ADP, but the enzyme activity of mutant K99A is lower than that of wild-type. This suggests that the RF loop may have two functions, not only helping ATP to bind at the active site, but also participating in the allosteric regulation of ADP.

In addition to the structure solved in this study, the reported PRPS structure bound with ADP in the allosteric site in PDB is only *Bacillus subtilis* PRPS (1DKU). Though the percent identity of hPRPS2 and *Bacillus subtilis* PRPS was 45%, the amino acids interacting with subtrates in active sites and ADP in allosteric sites were very conserved. The ATP active site in 1DKU binds to AMP, and AMP does not contain β phosphate, the residue R104 of *Bacillus subtilis* PRPS was located in the same position with residue K99 in hPRPS2, they may share same mod for ADP inhibition. Perhaps structures with ADP/GDP in the allosteric site 1 and ATP in the active site solved will help to test our hypothesis in the future.

### Filamentation and enzymatic activity

*E. coli* PRPS forms two types of filaments, type A attenuates ADP inhibition, and type B enhances the ADP inhibition. In this study, we found that the active form of hPRPS2 almost lost its activity when disrupting filament formation. In addition, hPRPS2 filament shares the same interface with *E. coli* PRPS type A filament. These key amino acid residues at the filament interface are conserved in humans, mice, flies, yeast and bacteria, indicating that filament regulation mechanisms may also exist in other species.

The structure comparison of hPRPS1 and hPRPS2 shows that they have the same amino acid residues at the filament interface. Previous study found rat PRPS1 and PRPS2, as well as the two so-called PAP-39 and PAP-41 peptides, can form big complex in rat liver [37]. During the preparation of this manuscript, Kollman and his colleagues reported in a preprint that hPRPS1 can form filaments [35]. We speculate that hPRPS1 and hPRPS2 may exist in the same filament. In the future, it will be interesting to solve the structure of the hPRPS1/hPRPS2 hybrid filament, if it exists.

### Purposes of filamentation

Filamentation of metabolic enzymes is very ubiquitous and conserved. The assembly of metabolic enzymes filament may affected by ligands binding, molecular crowding and pH [38, 39].Drosophila CTPS and human CTPS2 can form substrate-bound and product-bound filament [40–42], while human CTPS1 can only form substrate-bound filament, and *E.coli* CTPS can only form product-bound filament [43]. The polymerization of IMPDH octamers has been demonstrated to be regulated by its ligands, ATP, IMP and GTP. ATP and IMP promote the assembly of IMPDH filament, while GTP would destabilize the filament structure of IMPDH [44, 45].

Filamentation of metabolic enzymes can enhance activity, such as human IMPDH, CTPS and *Drosophila* CTPS [40, 43, 45]. Whereas filamentation of *E. coli* CTPS has an inhibition effect on activity. The filamentation of metabolic enzymes is not only a regulation method of enzyme activity, but has other function. It was reported that forming cytoophidia protects the protein from degradation [46, 47]. The expression level of *hPRPS2* increases in the metastasis and proliferation of many cancers, and decreased in the spermatogenesis deficiency and SCOS [30, 31]. Although it is not clear whether the reason for these phenomena is the change of PRPS2 activity or the change of protein itself, it will be very meaningful to study whether the formation of large-scale hPRPS2 filaments/bundles in vivo affects the half-life of hPRPS2.

In addition, mTOR pathway and post-translational modification of proteins have been proved to be related to the assembly of cytoophidia [48–52]. The study on PRPS2 also showed that the expression of *hPRPS2* driven by Myc interacted with mTOR, leading to tumor growth. Arginylation on N3 (asparagine) of PRPS2 affects its activity and stability [53]. Moreover, glucose deprivation leads to AMPK-mediated phosphorylation of PRPS1 and PRPS2 monomer associated with brain tumorigenesis [54].

It is attractive to study whether PRPS2 cytoophidia are related to mTOR pathway or affect post-translational modification. PRPS2 expression is associated with low spermatogenesis and SCOS through p53/Bcl-2/caspases signaling pathway. Another fascinating aspect is whether hPRPS cytoophidia, a large protein machinery formed by or in combination with PRPS2, affects biological function through other signaling pathway. Using our PRPS2 structure information, it will be easier to manipulate the activity and structure of PRPS2 in vivo through CRISPR genome engineering technique. As an important potential diagnostic and therapeutic method for Myc riven Cancers and male infertility, the application of genome engineering therapy in these serious diseases is worth studying.

In summary, our study solves the hPRPS2 filament structure with a resolution of 3.08 Å, in which ADP binds to the allosteric site and ATP active site in the hexamer. The key amino acid residues at the filament interface are conserved in humans, mice, flies and bacteraia. Disrupting filament formation of PRPS2 almost loses its activity. K99 in the RF loop may be a key amino acid residue related to the allosteric inhibition of ADP. Therefore, our work provides the basic structural information of PRPS2, and lays a foundation for studying the regulation of PRPS2 in the cell environment and its potential clinical application.

## Materials and methods

**Table:** 

Key Resources Table				
Reagent type (species) or resource	Designation	Source or reference	Identifiers	Additional information
Gene (*Drosophila melanogaster*)	PRPS	Genbank	P11908	
Strain, strain background (*Escherichia coli*)	Transetta (DE3)	TransGen Biotech		
Recombinant DNA reagent	pET28a-6His-SUMO	In house		
Commercial assay or kit	BCA Protein Concentration Determination Kit (Enhanced)	Beyotime	P0010	
Chemical compound, drug	Benzamidine hydrochloride	Sigma-Aldrich	434,760-5G	
Chemical compound, drug	Pepstatin A	Sigma-Aldrich	P5318-25MG	
Chemical compound, drug	Leupeptin hydrochloride microbial	Sigma/Aldrich	L9783-100MG	
Chemical compound, drug	PMSF	MDBio	P006-5 g	
Chemical compound, drug	Ni–NTA Agarose	QIAGEN	30,250	
Chemical compound, drug	Orotic acid	Adamas	01,102,798(74736A)	
Chemical compound, drug	ATP	Takara	4041	
Chemical compound, drug	D-Ribose 5 phosphate disodium salt	BIOSYNTH CARBOSYNTH	R-5600	
Chemical compound, drug	5-phospho-D-ribose 1-diphosphate penta-sodium salt	Sigma	P8296-25 mg	
Chemical compound, drug	Adenosine 5'-monophosphate	solarbio	A9860-1	
Chemical compound, drug	Adenosine 5'-diphosphate sodium salt	Sigma	A2754-100MG	
Other	Nitinol mesh	Zhenjiang Lehua Electronic Technology	M024-Au300-R12/13	Cryo-EM grid preparation
Other	Holey Carbon Film	Quantifoil	R1.2/1.3, 300 Mesh, Cu	Cryo-EM grid preparation
Other	400 mesh reinforced carbon support film	EMCN	BZ31024a	Negative staining
Software, algorithm	UCSF Chimera	10.1002/jcc.20084		https://www.cgl.ucsf.edu/chimera
Software, algorithm	UCSF Chimera X	10.1002/pro.3235		https://www.cgl.ucsf.edu/chimerax/
Software, algorithm	Relion	10.7554/eLife.42166		https://relion.readthedocs.io/en/latest/index.html#
Software, algorithm	Coot	10.1107/S0907444910007493		https://www2.mrc-lmb.cam.ac.uk/personal/pemsley/coot/
Software, algorithm	Phenix	10.1107/S2059798318006551		https://phenix-online.org/

### Human PRPS2 protein purification

Full-length wild-type or mutant human PRPS2 sequences with a C-terminal 6 × His-tag were cloned into a modified pRSFDuet vector and expressed in *E. coli* Transetta (DE3) cells. After induction with 0.1 mM IPTG at the OD_600_ range of 0.5 ~ 0.8, the cells were cultured at 37 °C for 4 h and pelleted by centrifugation at 4,000 r.p.m. for 10 min. The harvested cells were sonicated under the ice in lysis buffer (50 mM Tris–HCl pH 8.0, 500 mM NaCl, 10% glycerol, 20 mM imidazole, 1 mM PMSF, 5 mM β-mercaptoethanol, 5 mM benzamidine, 2 μg/ml leupeptin and 2 μg/ml pepstatin). After ultrasonication, the cell lysate was then centrifuged (15,000 r.p.m.) at 4 °C for 45 min. The supernatant was collected and incubated with equilibrated Ni–NTA agarose beads (Qiagen) for 1 h. and purified by Ni–NTA agarose beads (Qiagen). Lysis buffer with 50 mM imidazole was used to wash the column. And target proteins were eluted with lysis buffer with 250 mM imidazole. Further purification was performed in column buffer (25 mM Tris HCl pH 8.0 and 150 mM NaCl) using HiLoad Superdex 200 gel-filtration chromatography (GE Healthcare). The peak fractions were collected, concentrated, and stored in small aliquots at − 80 °C. All the experiments were performed at 4 °C.

### Negative staining

Wild-type or mutation hPRPS2 proteins were mixed with different substrate conditions. In brief, purified hPRPS2 protein (1 μM) was dissolved in Tris–HCl buffer (25 mM Tris–HCl, 150 mM NaCl, 10 mM MgCl_2_), and 2 mM ligands (ATP, ADP, AMP, R5P, or PRPP) was added to the solution. After incubation at 37 °C for 30 min, the prepared protein samples were applied to glow-discharged carbon-coated EM grids (400 mesh, EMCN), and stained with 1% uranyl acetate. Negative-stain EM grids were photographed on a Tecnai Spirit G21 microscope (FEI).

### Cryo-EM grid preparation and data collection

6 μM hPRPS2 protein was dissolved in a buffer containing 25 mM Tris HCl pH 7.5, 150 mM NaCl, 2 mM ADP, and 10 mM MgCl_2_ to generate filaments. The samples were incubated on ice for 15 min and then loaded on H_2_/O_2_ glow-discharged amorphous alloy film (CryoMatrix M024-Au300-R12/13). Then Grids were immediately blotted for 3.0 s with blot force of -1 and plunge-frozen in liquid ethane cooled by liquid nitrogen using Vitrobot (Thermo Fisher Scientific) at 4 °C and with 100% humidity.

Movies were recorded on Titan Krios G3 (FEI) equipped with a K3 Summit direct electron detector (Gatan), operating in counting super-resolution mode at 300 kV. Each movie stack was acquired in a total dose of 60 *e*^−^Å^−2^, subdivided into 50 frames at 4 s exposure. Automated data acquisition was performed with SerialEM [55] at a nominal magnification of 22,500 × and a calibrated pixel size of 1.06 Å, with defocus ranging from 1.0 to 2.5 μm.

### Image processing and 3D reconstruction

All image processing steps were performed using Relion3.1-beta [56]. MotionCor2 [57] and CTFFIND4 [58] were used to pre-process the image by RELION GUI. And the CTF (contrast transfer function) parameter was estimated by CTFFIND4. 681,672 particles were auto-picked from 2403 micrographs. After 2D classification, 458,154 particles were selected for 3D classification. After 3D classification using C1 and D3 symmetry, a total of 140,303 particles of the best category were selected for 3D auto-refinement, and each particle was subjected to CTF refinement and Bayesian polishing. we get an initial 3.3 Å density map including three layers of hPRPS2 hexamer. A final 3.08 Å map was sharpened by post-process using a tight mask for the central hexamer with a B-factor of 45 Å^2^.

### Model building and refinement

The structure of hPRP2 from AlphaFold was applied for the initial model. The hexamer models were generated and then docked into the corresponding electron density map by Chimera v.1.14. Coot [59]was used for iterative manual adjustment and rebuilding. The final atomic model was evaluated using MolProbity [60]. Real space refinements were performed with Phenix [61] The map reconstruction and model refinement statistics are listed in Supplementary Table 1. All figures were generated using UCSF Chimera [62] and ChimeraX [63].

### PRPS activity assay

The activity of hPRPS2 was measured by coupled continuous spectrophotometry using SpectraMax i3. The production PRPP of PRPS can be determined by a coupled reaction (OA + PRPP → OMP + PPi) of *E. coli* orotate phosphoribosyltransferase (OPRT, EC 2.4.2.10). The amount of PRPP generated in the reaction was determined by the reduction of orotate (OA) in the mixture. The concentration of OA was measured by absorbance at 295 nm for 300 s at 37°C [64]. Reaction mixture (100 μl) contains 0.5 μM PRPS, 1 mM OPRT, 1 mM OA, 10 mM MgCl_2_, 250 mM NaCl, 30 mM Na_2_HPO_4_, 1 mM R5P or ATP at concentrations as described in each experiment. ATP or R5P was least added into the mixture to initiate the reaction. All measurements were performed in triplicate.

## Supplementary Information

**Additional file 1: Figure S1.** Human PRPS2 is assembled into filaments in vitro. Negative staining electron microscopic images of purified human PRPS2 (1 μM) incubated in various conditions. The of nucleotides, phosphate ions (Pi) and Mg^2+^ are 2 mM, 30 mM and 10 mM, respectively. concentrations

**Additional file 2: Figure S2.** Cryo-EM data processing of human PRPS2 filament. **A** Representative Cryo-EM image of human PRPS2 filament. **B** Representative 2D averages of human PRPS2 filament in different views. **C** Local resolution of the type B filament final density map. **D** FSC curves of central hexamer in human PRPS2 filament density map (dash line shows FSC = 0.143). The final average resolution of hexamer is estimated to be 3.1 Å. **E** Flow chart of human PRPS2 filament image processing

**Additional file 3: Figure S3.** The medel of human PRPS2. **A** Cryo-EM reconstruction of human PRPS2 hexamer. The hexamer is the unit of filament. Each chain is in different color. **B** The reconstruction structure of human PRPS2 filament. The rise of human PRPS2 filament is 63 Å. When hexamers are aggregated into filament, the adjacent hexamer is twisted by 30°

**Additional file 4: Figure S4.** Catalytic activity of human PRS2 with different concentrations of ligands. Graphs show the catalytic activity of wild-type human PRPS2 in the presence of different amounts of ATP **A**, R5P **B**, and phosphate ion **C**. All tests are repeated three times.

**Additional file 5: Figure S5.** Structure comparison of PRPS. The structure comparison of hPRPS2 (colored in blue), E.coli PRPS type A filament (7XMU, colored in yellow), E.coli PRPS type A^AMP+ADP^ filament (7XMV, colored in green), E.coli PRPS type B filament (7XN3, colored in gray), Bacillus subtilis PRPS (1DKU, colored in magenta). Structure comparison of their hexamers (**A**), parallel dimmers (**B**), bent dimmers (**C**), monomers (**D**). The RMSD between hPRPS2 monomer and 7XMU, 7XMV, 7Xn3 monomer is 0.712, 0.742, 0.799, respectively

## Data Availability

Atomic models generated in this study have been deposited at the PDB under the accession codes 7YK1. Cryo-EM maps deposited to EMDB as: EMD-33883.
