# Supplementary figures and images for: Structural basis of human PRPS2 filaments
Source: Cell Biosci. 2023 May 30;13:100. doi: 10.1186/s13578-023-01037-z (PMC10227994; doi:10.1186/s13578-023-01037-z)

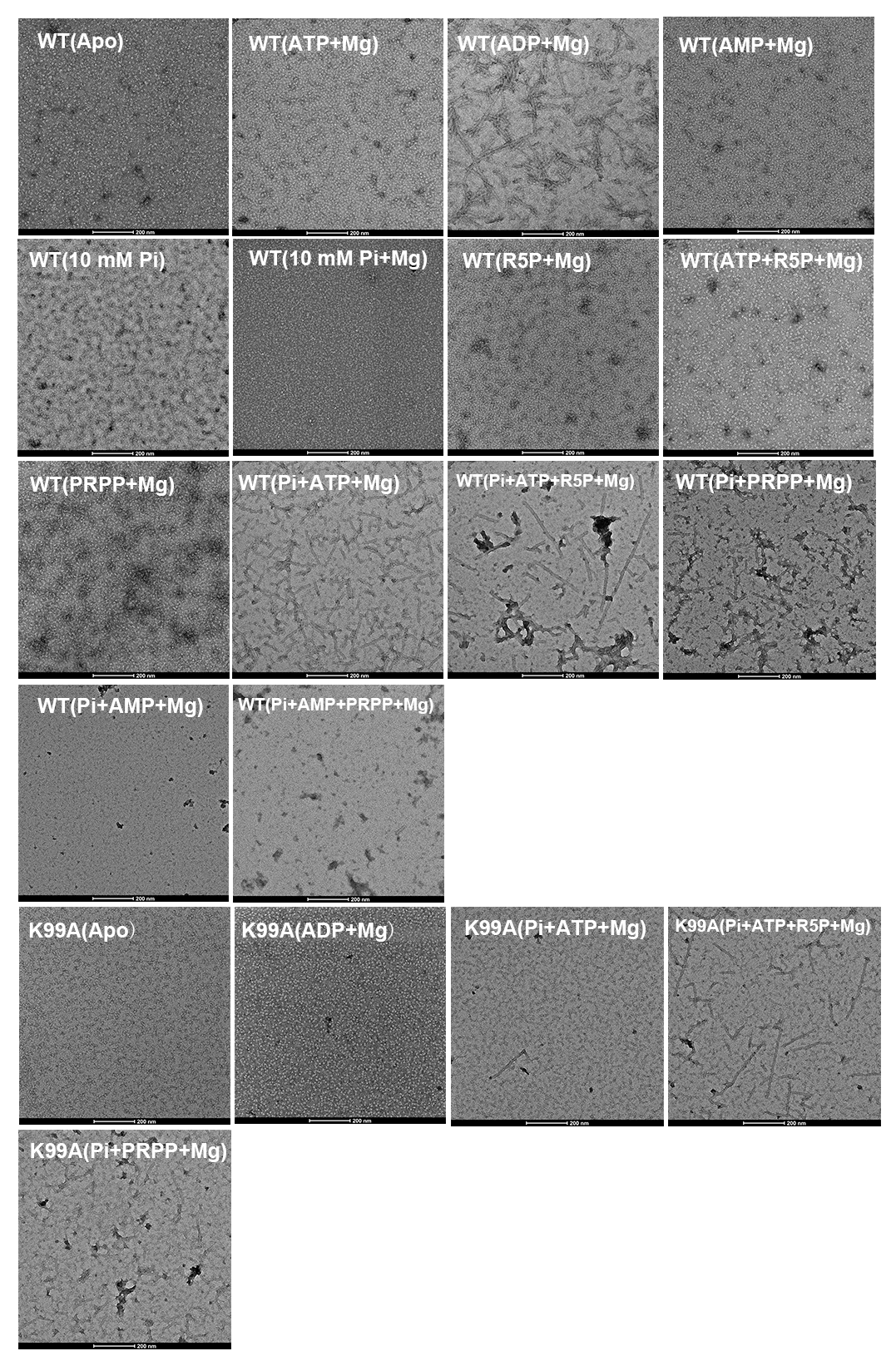

Supplement: Supplementary file 1 — Additional file 1: Figure S1. Human PRPS2 is assembled into filaments in vitro. Negative staining electron microscopic images of purified human PRPS2 (1 μM) incubated in various conditions. The of nucleotides, phosphate ions (Pi) and Mg2+ are 2 mM, 30 mM and 10 mM, respectively. concentrations [file 13578_2023_1037_MOESM1_ESM.tif]

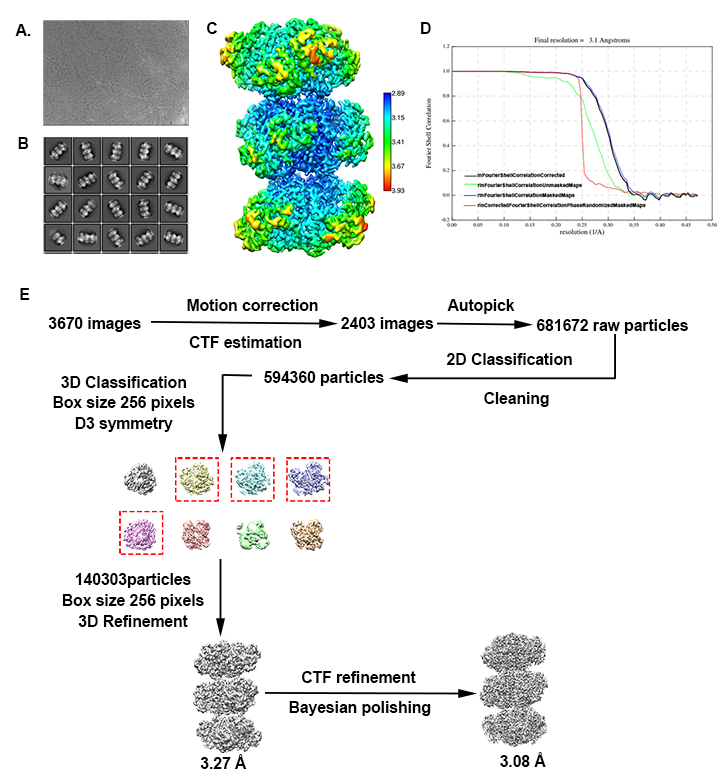

Supplement: Supplementary file 2 — Additional file 2: Figure S2. Cryo-EM data processing of human PRPS2 filament. A Representative Cryo-EM image of human PRPS2 filament. B Representative 2D averages of human PRPS2 filament in different views. C Local resolution of the type B filament final density map. D FSC curves of central hexamer in human PRPS2 filament density map (dash line shows FSC = 0.143). The final average resolution of hexamer is estimated to be 3.1 Å. E Flow chart of human PRPS2 filament image processing [file 13578_2023_1037_MOESM2_ESM.tif]

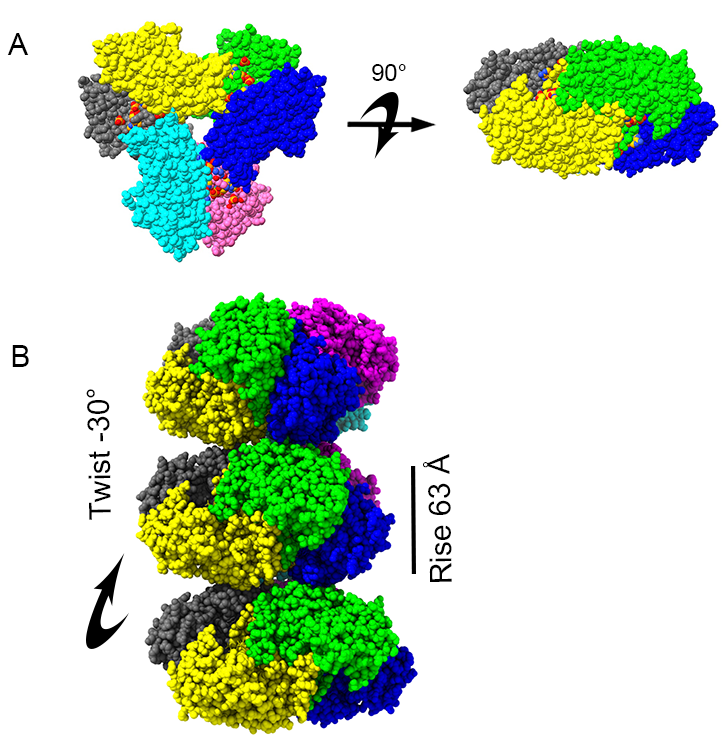

Supplement: Supplementary file 3 — Additional file 3: Figure S3. The medel of human PRPS2. A Cryo-EM reconstruction of human PRPS2 hexamer. The hexamer is the unit of filament. Each chain is in different color. B The reconstruction structure of human PRPS2 filament. The rise of human PRPS2 filament is 63 Å. When hexamers are aggregated into filament, the adjacent hexamer is twisted by 30° [file 13578_2023_1037_MOESM3_ESM.tif]

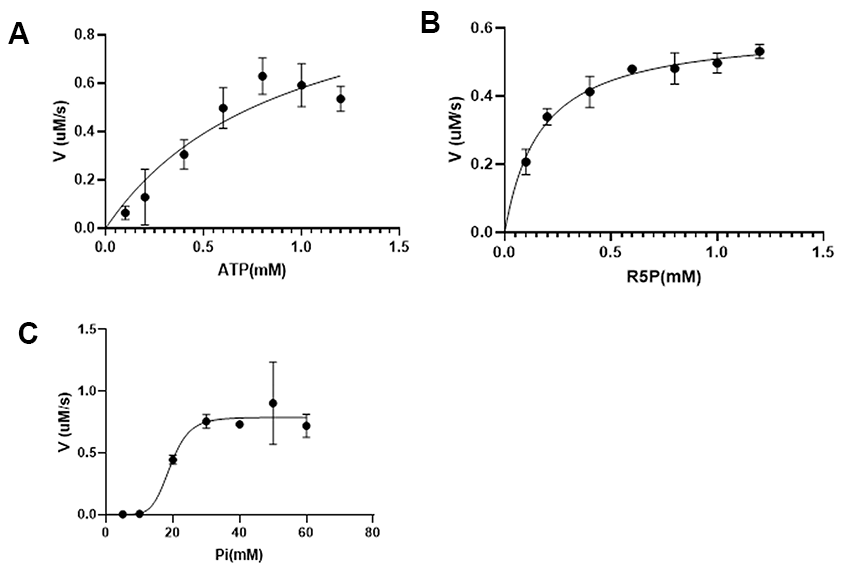

Supplement: Supplementary file 4 — Additional file 4: Figure S4. Catalytic activity of human PRS2 with different concentrations of ligands. Graphs show the catalytic activity of wild-type human PRPS2 in the presence of different amounts of ATP A, R5P B, and phosphate ion C. All tests are repeated three times. [file 13578_2023_1037_MOESM4_ESM.tif]

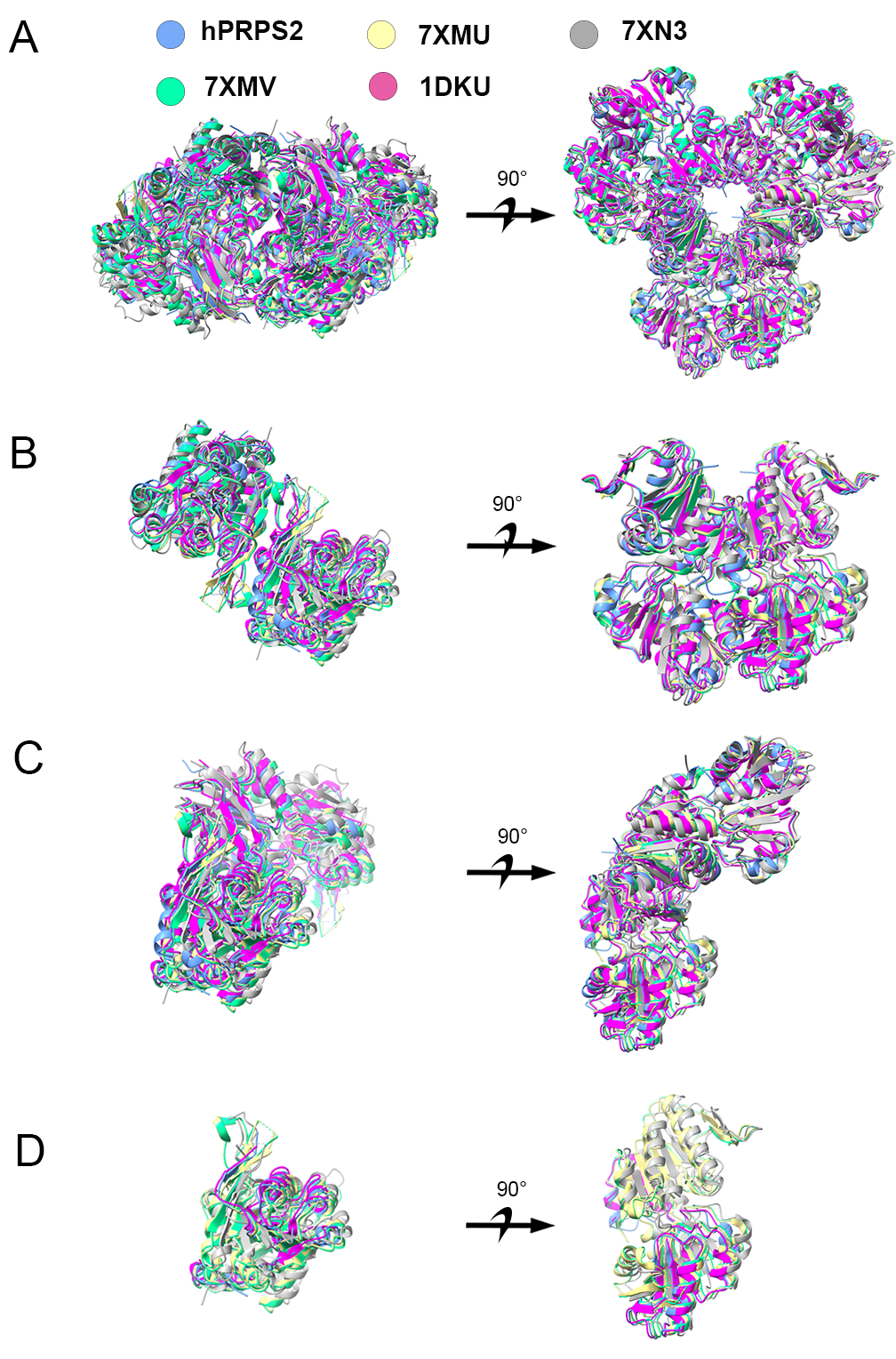

Supplement: Supplementary file 5 — Additional file 5: Figure S5. Structure comparison of PRPS. The structure comparison of hPRPS2 (colored in blue), E.coli PRPS type A filament (7XMU, colored in yellow), E.coli PRPS type AAMP+ADP filament (7XMV, colored in green), E.coli PRPS type B filament (7XN3, colored in gray), Bacillus subtilis PRPS (1DKU, colored in magenta). Structure comparison of their hexamers (A), parallel dimmers (B), bent dimmers (C), monomers (D). The RMSD between hPRPS2 monomer and 7XMU, 7XMV, 7Xn3 monomer is 0.712, 0.742, 0.799, respectively [file 13578_2023_1037_MOESM5_ESM.tif]
